# Transcatheter arterial embolisation is efficient and safe for paediatric blunt torso trauma: a case-control study

**DOI:** 10.1186/s12873-020-00381-4

**Published:** 2020-10-31

**Authors:** Masayasu Gakumazawa, Chiaki Toida, Takashi Muguruma, Naoki Yogo, Mafumi Shinohara, Ichiro Takeuchi

**Affiliations:** Department of Emergency Medicine, Yokohama City University Graduate School of Medicine, 4-57 Urafunecho, Minami-ku, Yokohama, 232-0024 Japan

**Keywords:** Paediatric patients, Trauma care, Nonsurgical intervention, Intervention radiology, Transcatheter arterial embolisation, Complication, In-hospital mortality, Standardised mortality ratio

## Abstract

**Background:**

It remains unclear whether transcatheter arterial embolisation (TAE) is as safe and effective for paediatric patients with blunt torso trauma as it is for adults in Japan, owing to few trauma cases and sporadic case reports. The study aimed to compare the efficacy and safety of TAE performed in paediatric (age ≤ 15 years) and adult patients with blunt torso trauma.

**Methods:**

This was a single-centre, retrospective chart review study that included blunt torso trauma patients who underwent TAE in the trauma centre from 2012 to 2017. The comparative study was carried out between a ‘paediatric patient group’ and an ‘adult patient group’. The outcome measures for TAE were the success of haemorrhage control and complications and standardised mortality ratio (SMR).

**Results:**

A total of 504 patients with blunt torso trauma were transported to the trauma centre, out of which 23% (*N* = 114) with blunt torso trauma underwent TAE, including 15 paediatric and 99 adult patients. There was no significant difference between the use of TAE in paediatric and adult patients with blunt torso trauma (29% vs 22%, *P* = .221). The paediatric patients’ median age was 11 years (interquartile ranges 7–14). The predicted mortality rate and SMR for paediatric patients were lower than those for adult patients (18.3% vs 25.9%, *P* = .026, and 0.37 vs 0.54). The rate of effective haemorrhage control without repeated TAE or additional surgical intervention was 93% in paediatric patients, which was similar to that in adult patients (88%). There were no complications in paediatric patients at our centre. There were no significant differences in the proportion of paediatric patients who underwent surgery before TAE or urgent blood transfusion (33% vs 26%, *P* = .566, or 67% vs 85%, *P* = .084).

**Conclusions:**

It is possible to provide an equal level of care related to TAE for paediatric and adult patients as it relates to TAE for blunt torso trauma with haemorrhage in the trauma centre. Alternative haemorrhage control procedures should be established as soon as possible whenever the patients reach a haemodynamically unstable state.

## Background

Although torso trauma with excessive bleeding has been associated with significant morbidity and mortality, the selection of the therapeutic strategy for haemodynamically unstable patients remains a challenge [1–3]. Due to advances in endovascular techniques for trauma patients, the therapeutic strategy in blunt torso trauma with haemorrhage changed from operative to non-operative management in the mid-1990s [4, 5]. In adult patients, non-operative management has been established as a standard of care for trauma patients who are haemodynamically stable [3, 4, 6]. Previous studies reported transcatheter arterial embolisation (TAE) as one of the non-operative management strategies to improve morbidity and mortality for blunt torso trauma patients with acute bleeding [3, 5, 6].

Conversely, children have age-dependent anatomical and physiological differences and a relatively low incidence of blunt torso trauma [7, 8]. A previous study on children cohort reported that non-operative management for haemodynamically stable paediatric patients with blunt torso trauma was the gold standard of trauma care [7, 9]. Most paediatric patients are now managed with observation. Paediatric patients who undergo additional therapeutic interventions, such as blood transfusion, TAE, or surgery are uncommon [7, 9]. However, to the best of our knowledge, there is no research that evaluates the best management strategy, including non-operative and operative interventions, for haemodynamically unstable paediatric patients with blunt torso trauma. It remains unclear whether TAE for paediatric patients with blunt torso trauma is as effective and safe as that for adults, due to the relatively few trauma centres and sporadic case reports [10, 11]. The aim of this study was to evaluate the efficacy and safety of TAE for paediatric patients with blunt torso trauma by comparing them with adult patients in Yokohama City University Medical Centre (Yokohama, Japan), which has adapted the same therapeutic algorithm in blunt torso patients with haemorrhage regardless of age.

## Methods

### Study setting and population

This was a single centre study, conducted retrospectively in Yokohama City University Medical Centre (Yokohama, Japan). Our centre is one of the two Yokohama City Major Trauma Centres (YCMTCs), which were established to serve a population of 3.7 million, including 446,000 children, and to provide 24/7 trauma care by a specialised team, including an interventional radiologist, on-call around the clock [12, 13]. Before severe trauma patients arrive at our centre, an in-hospital trauma code is activated and preparations for blood transfusions, urgent surgery, and interventional radiology (IVR) are initiated. Whenever trauma patients are in an unstable condition, we can perform urgent surgical and/or radiological interventions within the first 30–60 min of arrival to hospital.

**Fig. 1:**
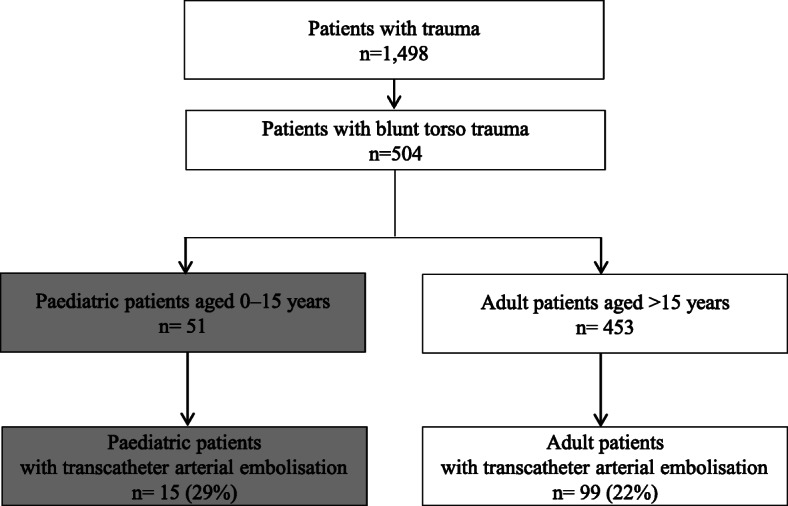
Flow diagram of the study population

For this study, we used the dataset from our centre to include information between January 1, 2014, and December 31, 2017, which initially yielded the data for 19,207 patients. The inclusion criteria for this study were: blunt torso trauma patients and patients who underwent TAE. Patients who were dead on arrival were excluded from this study. Figure 1 presents a flow chart of the patient population in this study.

### Emergency department algorithm in blunt torso patients with haemorrhage at YCMTC

All trauma patients underwent evaluation and trauma care in the acute care phase, according to the Japanese trauma evaluation and care guidelines [14]. The general approach is based on the patients’ haemodynamic stability and response to fluid resuscitation. If systolic blood pressure is less than 90 mmHg in an adult and less than the age-related baseline value in children [15], these patients are considered to be in hypovolemic shock.

Regarding fluid resuscitation, 20 ml of crystalloid solution per body weight (kg) is rapidly administered, followed by whole blood transfusion. First, if the patients do not respond to fluid resuscitation but achieve haemodynamic stability, they are assessed as ‘non-responders’ and treated by surgical intervention with damage control techniques. In case of persistent haemodynamic instability with ongoing arterial bleeding, subsequent TAE is performed. Second, if the patient responds to fluid resuscitation and maintains haemodynamic stability, the patient is assessed to be a ‘responder’ and will undergo contrast-enhanced computed tomography (CT) scan. In case of arterial extravasation, TAE is performed. Finally, if the patient responds to fluid resuscitation and achieves haemodynamic stability at least temporarily, they are assessed to be ‘transient-responder’ and undergo contrast-enhanced CT scan. According to the patient’s condition and the results of the CT scan, the trauma team will perform TAE and/or surgical intervention for the transient-responders.

All blunt trauma patients with haemorrhage in this study underwent trauma care according to our institutional algorithm, as aforementioned. Therefore, the trauma team performed operative and/or non-operative intervention, using the same therapeutic strategy, regardless of age; however, in the case of paediatric patients, all interventions were performed under the management of general anaesthesia and mechanical ventilation. The technique for TAE started with percutaneous femoral artery vascular access with sheath placement. Arterial puncture in younger paediatric patients used an ultrasonically guided puncture needle to ensure safety and reliable manual operation. A 4-, 5-, or 6-Fr sheath was used to maintain arterial access throughout the procedure. Finally, all image diagnoses and IVR were performed by the interventional radiologist. Various materials are used for TAE, broadly categorised as permanent (coils or n-butyl-2-cyanoacrylate) or temporary (gelatin particles). The method of following up paediatric patients who underwent TAE was as follows: (a) an emergency physician confirmed that the patients are in a haemodynamic stability state and removed the arterial sheath after 6 or 12 h of TAE; (b) before and after TAE, the patients, with a pressure band, rested on the bed for 6 h; (c) an emergency physician evaluated whether the patients have complications, such as puncture site hematomas or arterial embolism; and (d) an emergency physician also evaluated whether the patients had complications, such as pseudoaneurysm or arteriovenous fistulas on injured organs, using ultrasound or CT scan approximately 10 days after injury.

### Data collection and outcome measurements

We collected the following information: age (years), body weight, mechanism of injury, transportation method, vital signs and haemodynamic state on hospital arrival, Injury severity score (ISS) [16], Revised trauma score (RTS) [17], predicted survival rate (%) calculated by using the trauma and injury severity score (TRISS) [18], provision of an urgent examination and treatment during the acute care phase, duration of mechanical ventilation (days), intensive care unit (ICU) stay (days), and hospital stay (days), in-hospital mortality rate (%), standardised mortality ratio (SMR), TAE data including treatment before IVR, time interval from arrival to the beginning of IVR, embolic agents, target region and artery of embolisation, degree of haemorrhage control and complication rate. The SMR was calculated by dividing the in-hospital mortality rate by the mean predicted mortality rate.

The outcome measures for TAE were the success of haemorrhage control and complications following embolisation, the need for surgical intervention or repeat embolisation, and SMR. The degree of haemorrhage control was classified as follows: (a) effective haemorrhage control; (b) ongoing haemorrhage; and (c) exsanguination and death [8]. Complications were classified as major and minor using the Society of Interventional Radiology classification system [19].

### Data analysis

The patients enrolled in this study were categorised into a ‘paediatric patient group’ (younger than 15 years) and an ‘adult patient group’ (older than 15 years). The age threshold of 15 years set by Basis Education Law, at which Japanese children must receive compulsory education was chosen to classify patients as either children or adults. The results of these comparisons are expressed as the medians and interquartile ranges (IQRs) [25th–75th percentile] for continuous variables and as the means and percentages for categorical variables. The Mann–Whitney *U* test and Kruskal–Wallis tests were used to analyse the continuous variables, whereas Fisher’s exact test was used for the categorical variables. All statistical analyses were carried out by using STATA/SE software, version 16.0 (StataCorp; College Station, Texas, USA). A two-tailed *P-*value of < 0.05 indicated statistical significance.

## Results

During the 6-year study period, of the 19,207 patients observed at our centre, 1498 were trauma patients who were transported by the emergency medical service, including 504 patients with blunt torso trauma. Approximately 23% (*N* = 114) of all patients with blunt torso trauma underwent TAE. This study population included 15 paediatric and 99 adult patients. There was not a significant difference between the incident rate of TAE for paediatric and adult patients with blunt torso trauma (29% vs 22%, *P* = .221; Fig. 1). The median age was 43 (IQR 22–60). Moreover, 52% of all participants were injured in traffic accidents.

Table 1 presents a comparison of the characteristics and outcome in paediatric and adult patients who underwent TAE for blunt torso trauma. The median age was 11 years old (IQR 7–14) for paediatric patients and 47 years old (IQR 34–64) for adults (*P* < .001). Compared with the adult patients, a higher proportion of paediatric patients were transported from other hospitals (67% vs 12%, *P* < .001). The median RTS of paediatric patients was higher than adult patients [7.84 (IQR 5.82–24) vs 6.61 (IQR 5.56–7.55), *P* = .031]. The predicted mortality rate and SMR for paediatric patients were lower than that for adult patients (18.3% vs 25.9%, *P* = .025, and 0.37 vs. 0.54).

**Table 1:** Characteristics and outcome compared between paediatric and adult trauma patients with transcatheter arterial embolisation

Variable	All patients	Paediatric patients	Adult patients	*P*-value
	(*n* = 114)	(*n* = 15)	(*n* = 99)	
Male, n (%)	79 (69)	11 (73)	68 (69)	0.716
Age in year, (median, IQR)	43 (22–60)	11 (7–14)	47 (34–64)	<0.001
Body weight, kg, (median, IQR)	61 (53–69)	39.7 (24.1–52.1)	63.2 (54.6–69.6)	<0.001
Mechanism of injury, n (%)				
Traffic accident	59 (52)	7 (47)	52 (53)	0.700
Fall from a height	48 (42)	5 (33)	43 (43)	0.460
Fall at same place	1 (0.9)	1 (7)	0	0.010
Unspecified	6 (5)	2 (13)	4 (4)	0.133
Transportation from other hospitals, n (%)	22 (19)	10 (67)	12 (12)	<0.001
Injury severity score, (median, IQR)	29 (17–41)	26 (10–41)	29 (18–41)	0.719
Revised trauma score, (median, IQR)	6.66 (5.68–7.84)	7.84 (5.82–7.84)	6.61 (5.56–7.55)	0.031
Duration of mechanical ventilation, days, (median, IQR)	9 (3–15)	4 (2–16.5)	9 (3–15)	0.468
Duration of ICU stay, days, (median, IQR)	10 (4–16)	6 (3–16)	11 (4–16)	0.438
Duration of hospital stay, days, (median, IQR)	34 (13–63)	19 (12–52)	35 (13–66)	0.303
In-hospital actual mortality rate, (%)	13.2	6.7	14.1	0.165
Predicted mortality rate, (%)	24.9	18.3	25.9	0.026
Standardised mortality ratio	0.53	0.37	0.54	–

*IQR* Interquartile range, *ICU* intensive care unit

Table 2 presents a comparison of the vital signs and blood-examination upon arrival to the hospital between the paediatric and adult patients. With regard to the haemodynamic stability, there were no differences in the proportion of the patients classified as ‘non-responder’, ‘transient-responder’, and ‘responder’. When compared with adult patients, the median Glasgow Coma Scale was higher [15 (IQR 8–15) vs 13 (IQR 7–14), *P* = .046], the median haemoglobin value was higher [11.9 g/dL (IQR 9.4–11.9) vs 12.2 (IQR 10.9–13.5), *P* = .006], the median base excess was higher [− 0.9 mmol/L (IQR -2.9–-0.9) vs. -4.0 (IQR -6.7–-1.8), *P* = .001], and the median lactate value was lower [2.3 mg/dL (IQR 1.3–3.2) vs. 3.7 (IQR 2.5–5.9), *P* = .004] in paediatric patients. There are no significant differences in the duration of mechanical ventilation/ICU stay/hospital stay (Table 1), the proportion of urgent blood transfusion, time interval from the arrival to blood transfusion, and dosage of blood transfusion (Table 3).

**Table 2:** Vital sign/blood examination between paediatric and adult trauma patients with transcatheter arterial embolisation upon arrival

Variable	All patients	Paediatric patients	Adult patients	*P*-value
	(*n* = 114)	(*n* = 15)	(*n* = 99)	
Patients with hypovolemic shock	104 (91)	12 (80)	92 (93)	0.099
Non-responder	16 (14)	2 (13)	14 (14)	0.933
Transient-responder	60 (53)	6 (40)	54 (55)	0.293
Responder	28 (25)	4 (27)	24 (24)	0.839
Vital sigh at hospital arrival				
Systolic blood pressure, mmHg, (median, IQR)	109 (84–136)	122 (107–133)	108 (82–141)	0.209
Diastolic blood pressure, mmHg, (median, IQR)	66 (50–85)	65 (59–73)	67 (46–87)	0.668
Heart rate, bpm, (median, IQR)	105 (84–124)	113 (102–121)	103 (81–126)	0.323
Respiratory rate, /min, (median, IQR)	23 (18–28)	20 (20–27)	23 (17–30)	0.759
Glasgow Coma Scale, (median, IQR)	13 (7–15)	15 (8–15)	13 (7–14)	0.046
Body temperature, °C, (median, IQR)	36.4 (35.8–36.8)	37.1 (36.9–37.3)	36.3 (35.8–36.7)	< 0.001
Blood examination at hospital arrival				
Leucocytes, /μL, (median, IQR)	13,010 (8760–17,513)	13,860 (10810–17,230)	12,580 (8720–17,675)	0.431
Hemoglobin, g/dL, (median, IQR)	11.9 (10.8–13.3)	11.1 (9.4–11.9)	12.2 (10.9–13.5)	0.006
Platelet, /μL, (median, IQR)	20.8 (17.1–24.4)	19.5 (15.0–26.7)	20.9 (17.2–24.2)	0.988
Fibrinogen, mg/dL, (median, IQR)	213 (170–264)	166 (140–311)	218 (177–263)	0.416
Fibrinogen/fibrin degradation products, μg/mL, (median, IQR)	97.5 (26.4–169.1)	48.2 (12.6–225.4)	98.2 (27.4–164.2)	0.729
D-dimer, μg/mL, (median, IQR)	48.2 (17.6–95.7)	27.0 (6.7–127.2)	53.4 (20.2–94.3)	0.405
Base excess, mmol/L, (median, IQR)	−3.7 (−6.3– –1.3)	−0.9 (−2.9–0.9)	−4.0 (−6.7– –1.8)	0.001
Lactate, mg/dL, (median, IQR)	3.5 (2.3–5.6)	2.3 (1.3–3.2)	3.7 (2.5–5.9)	0.004

*IQR* Interquartile range, *RBC* red cell concentrate, *FFP* fresh frozen plasma

**Table 3:** Blood transfusion comparison in paediatric and adult trauma patients with transcatheter arterial embolisation

Variable	All patients	Paediatric patients	Adult patients	*P*-value
	(*n* = 114)	(*n* = 15)	(*n* = 99)	
Blood transfusion during primary trauma care, n (%)	94 (83)	10 (67)	84 (85)	0.084
RBC	89 (78)	10 (67)	79 (80)	0.252
FFP	91 ((80)	10 (67)	81 (82)	0.173
Platelets	30 (26)	1 (7)	29 (29)	0.051
Time interval from hospital arrival to beginning of blood tranfusion, minutes, (median, IQR)				
RBC	40 (23–78)	56 (34–78)	40 (22–77)	0.441
FFP	84 (56–111)	91 (47–113)	84 (57–111)	0.874
Platelets	143 (115–160)	78 (78–78)	145 (119–161)	0.067
Dosage of blood transfusion, International Units per body weight, (median, IQR)				
RBC	0.1 (0.03–0.2)	0.10 (0–0.2)	0.1 (0.04–0.2)	0.919
FFP	0.2 (0.04–0.2)	0.1 (0–0.2)	0.2 (0.1–0.2)	0.488
Platelets	0 (0–0)	0 (0–0)	0 (0–0.2)	0.133

*IQR* Interquartile range, *RBC* red cell concentrate, *FFP* fresh frozen plasma

Table 4 presents a summary of 114 patients who underwent TAE. Although there were no differences in the time interval from the arrival to IVR, the median time interval from the beginning to the end of IVR was significantly shorter [55 (IQR 43–59) vs. 65 (IQR 51–76) minutes, *P* = .007]. With regard to the target region and artery embolised, the proportion of paediatric patients who had undergone intra-pelvic arterial embolisation was lower (27% vs 85%, *P* < .001, and 27% vs 71%, *P* = .001). There were no significant differences in the embolic agents, degree of haemorrhage control, or the proportion of patients with repeated IVR and complication rate. Although no paediatric patient had repeated IVR and complications, two adult patients underwent repeated IVR, and three patients experienced complications including two major complications (deviation of coil/necrosis of gluteus muscle), and one minor complication (self-limiting puncture site hematomas).

**Table 4:** Summary of intervention radiology compared between paediatric and adult trauma patients with transcatheter arterial embolisation

Variable	All patients	Paediatric patients	Adult patients	*P* value
	(*n* = 114)	(*n* = 15)	(*n* = 99)	
Treatment before IVR, n (%)				
Resuscitative endovascular balloon of the aorta	4 (4)	1 (7)	3 (3)	0.476
Surgical intervention	31 (27)	5 (33)	26 (26)	0.566
Time interval, minutes, (medican, IQR)				
from arrival to beginning of IVR	89 (72–120)	78 (66–132)	89 (74–119)	0.299
from beginning to end of IVR	66 (48–73)	55 (43–59)	65 (51–76)	0.007
Size of catheter sheath, French Gauge, (median, IQR)	6 (5–6)	5 (4–5)	6 (5–6)	< 0.001
Embolic agents, n (%)				
Coils	49 (43)	6 (40)	43 (43)	0.802
Gelatin particles	102 (90)	13 (87)	89 (90)	0.704
NBCA	71 (62)	7 (47)	64 (65)	0.181
Target region of embolisation, n (%)				
Chest and/or abdominal region	99 (87)	13 (87)	86 (87)	0.983
Pelvic region	88 (77)	4 (27)	84 (85)	< 0.001
Target artery of embolisation, n (%)				
Internal thoracic artery	1 (1)	0	1 (1)	0.696
Intercostal artery	6 (5)	3 (20)	3 (3)	0.006
Phrenic artery	6 (5)	1 (7)	5 (5)	0.794
Splenic artery	21 (18)	5 (33)	16 (16)	0.110
Hepatic artery	20 (18)	4 (27)	16 (16)	0.319
Renal artery	10 (9)	1 (7)	9 (9)	0.757
Adrenal artery	7 (6)	3 (20)	4 (4)	0.016
Colonic artery	2 (2)	0	2 (2)	0.579
Superio mesenteric artery	1 (1)	0	1 (1)	0.697
Inferio mesenteric artery	2 (2)	0	2 (2)	0.579
Gastroduodenal artery	1 (1)	0	1 (1)	0.697
Pelvic artery	75 (66)	4 (27)	71 (71)	0.001
Lumbar artery	48 (42)	4 (27)	44 (44)	0.194
Degree of haemorrhage control, n (%)				
Effective haemorrhage control	101 (89)	14 (93)	87 (88)	0.536
Ongoing haemorrhage	9 (8)	1 (7)	8 (8)	0.850
Exsanguination and death	4 (4)	0	4 (4)	0.428
Repeated IVR, n (%)	4 (4)	0	4 (4)	0.428
Patients with complications, n (%)				
Major	2 (2)	0	2 (2)	0.579
Minor	1 (1)	0	1 (1)	0.696

*IQR* Interquartile range, *TAE* transcatheter arterial embolisation, *IVR* interventional radiology, *NBCA* n-butyl-2-cyanoacrylate

A detailed summary of the 15 paediatric patients who underwent TAE for blunt torso trauma is shown in Table 5. The median transfer time from injury to arrival at our hospital was longer for patients transported from another hospital than that for patients transported from the site of injury (274.5 min vs 43 min, *P* < 0.05). Two paediatric patients who were classified as ‘non-responder’ underwent surgical intervention and/or resuscitative endovascular balloon occlusion before the IVR. Two of the six paediatric patients classified as ‘transient-responder’ underwent surgical intervention before the IVR. One of the paediatric patients classified as ‘responder’ underwent surgical intervention in a different target region of TAE. The proportion of paediatric patients classified as having non-effective haemorrhage control (ongoing haemorrhage) after TAE and underwent additional surgical intervention was 7%.

**Table 5:** Detailed summary of paediatric patients with transcatheter arterial embolisation

Case	Age, year	Mechanism of injury	Transportation method	Total transfer time from injury to arrival at our hospital, minutes	Target region of embolisation	Revised trauma score	Injury severity score	Predicted survival rate, %	Patients with hypovolemic shock	Treatment before IVR	Size of catheter sheath, French	Embolic agents	Time interval from arrival to beginning of IVR, minutes	Time interval from beginning to end of IVR, minutes	Degree of haemorrhage control	IVR repeated	Complication	ICU stay, days	Hospital stay, days	Outcome
1	4	Fall from a height	From another hospital	276	Chest / abdominal region	7.55	9	99.3	Transient-responder	None	5	Coils	48	61	Effective control	None	None	6	9	Alive
2	5	Fall from a height	From the site of injury	48	Chest / abdominal region	7.84	16	99.4	Non-shock	None	4	Gelatin particles, Coils	149	26	Effective control	None	None	3	23	Alive
3	5	Fall from a height	From another hospital	293	Chest / abdominal region	2.34	59	3.0	Non-responder	Surgical intervention	4	Gelatin particles, NBCA	90	60	Effective control	None	None	70	109	Alive
4	7	Traffic accident	From another hospital	140	Pervic region	7.84	26	97.6	Transient-responder	None	5	Gelatin particles	68	45	Effective control	None	None	17	58	Alive
5	7	Traffic accident	From the site of injury	26	Chest / abdominal / pervic region	4.09	43	32.5	Responder	Surgical intervention	4	Gelatin particles, Coils、NBCA	785	55	Effective control	None	None	8	19	Death
6	8	Fall at same place	From another hospital	273	Chest / abdominal region	7.84	9	99.6	Non-shock	None	5	Gelatin particles, Coils	38	42	Effective control	None	None	3	9	Alive
7	9	Traffic accident	From another hospital	215	Chest / abdominal region	7.84	26	97.6	Transient-responder	None	4	Gelatin particles	57	67	Effective control	None	None	6	13	Alive
8	11	Traffic accident	From another hospital	214	Pelvic region	7.84	9	99.4	Responder	None	5	Gelatin particles	69	57	Effective control	None	None	1	6	Alive
9	11	Fall from a height	From another hospital	281	Chest / abdominal / pervic region	7.84	34	99.5	Responder	None	5	Gelatin particles, NBCA	71	57	Effective control	None	None	4	46	Alive
10	12	Unspecified	From another hospital	113	Chest / abdominal region	7.84	27	94.9	Responder	None	5	Gelatin particles, NBCA	89	53	Effective control	None	None	4	19	Alive
11	14	Traffic accident	From another hospital	432	Chest / abdominal region	7.84	10	99.4	Non-shock	None	6	Gelatin particles, Coils	64	24	Effective control	None	None	3	11	Alive
12	14	Unspecified	From another hospital	3 days	Chest / abdominal region	7.11	9	98.9	Transient-responder	None	5	Gelatin particles, Coils	72	46	Ongoing haemorrhage	None	None	3	15	Alive
13	14	Traffic accident	From the site of injury	45	Chest / abdominal region	5.97	50	76.9	Transient-responder	Surgical intervention	5	Gelatin particles, NBCA	162	35	Effective control	None	None	27	73	Alive
14	15	Traffic accident	From the site of injury	39	Chest / abdominal region	5.03	41	60.9	Transient-responder	Surgical intervention	5	Gelatin particles, NBCA	116	59	Effective control	None	None	23	42	Alive
15	15	Fall from a height	From the site of injury	43	Chest / abdominal region	5.68	41	67.2	Non-responder	REBOA, Surgical intervention	6	NBCA	180	61	Effective control	None	None	15	64	Alive

*REBOA* Resuscitative endovascular balloon occlusion of the aorta, *IVR* interventional radiology, *NBCA* n-butyl-2-cyanoacrylate

## Discussion

This study evaluated the efficacy and safety of TAE for blunt trauma patients at Yokohama City University Medical Centre. Applying the same diagnostic and therapeutic algorithm for blunt torso trauma patients with haemorrhage regardless of age, TAE might be equally effective and safe for paediatric blunt torso trauma in comparison to adult patients.

Since non-operative management has become the standard of care for blunt solid organ injury in children, the incidence of TAE for paediatric patients with blunt abdominal or pelvic trauma varies from 1.4 to 2.1% in a previous study [7, 8, 20, 21]. Moreover, there are very few studies on the incidence rate of TAE for blunt torso paediatric patients in comparison to adult patients. In patients with pelvic fracture, the incidence rate of IVR was not significantly different between paediatric and adult patients (2.1% vs 4.8%) [20]. This study also showed the different results, as the incident rate of TAE for blunt torso trauma patients was higher than those of previous study, and similar results as the incident rate of TAE for blunt torso trauma patients was similar in paediatric and adult patients (29% vs 22%, *P* = .221; Fig. 1). The reason why there are big differences in the incident rate of TAE for paediatric patients with torso trauma between this study and previous studies, and may be related to the association of the incident rate with varying severity and complexity of injuries in study cohort [7]. However, the number of paediatric patients who underwent TAE was extremely low compared to that of adults in both studies, as the incidence of severe trauma was limited. There were only 15 paediatric patients who underwent TAE during the 6-year study period of the current study. A previous study showed that hospitals with a high-volume of admitted patients and therapeutic experience can provide high-quality of care and lead to lower mortality rate for severe trauma patients [22]. To improve the outcome of TAE for torso trauma patients with haemorrhage, further centralisation of trauma patients might be effective. In this study, 67% of all the paediatric patients who underwent TAE were transferred from another hospital and the transfer time from injury to arrival at our hospital was longer for these patients than that for patients transported to our hospital directly from the site of injury. Therefore, an appropriate transport protocol for long-distance or inter-hospital transportation might be effective to improve the outcome of blunt torso patients with haemorrhage, especially in paediatric patients.

With regard to efficacy in this study, the rate of effective haemorrhage control without repeated TAE or additional surgical intervention was 93% for all paediatric patients, which was similar to that for adult patients (88%). The success rate of TAE was also similar to the results of previous studies that reported a 75–100% success rate of TAE in paediatric cohorts [8, 20, 21] and 77–100% success rate in cohorts with paediatric and adult patients [5, 6]. Although we cannot compare the mortality rate directly between the paediatric and adult patients because there are differences in the injury site and severity between two groups, we found that the SMR of paediatric patients was lower than that of adults in this study. These results suggested that trauma centres that do not specialise in paediatric patients could provide TAE safely and effectively for adult and paediatric patients.

There were no complications in paediatric patients, when we used the same diagnostic and therapeutic algorithm for blunt torso trauma patients with a haemorrhage for all ages at our centre. Moreover, the complication rate in this study was similar to that reported in previous studies (i.e., 0–7%) [4–8]. With regard to additional urgent therapy before and after the TAE, there were no significant differences in the proportion of patients who underwent an urgent blood transfusion, resuscitative endovascular balloon occlusion of the aorta (REBOA), or surgical intervention in this study between paediatric and adult patients. Although a previous study reported that few paediatric patients with blunt torso trauma needed the urgent intervention, such as blood transfusion or surgical intervention [7, 9], our results showed that there were not a few paediatric patients with blunt torso trauma who urgently need alternative haemorrhage control procedures before and after TAE. This result suggested that TAE is a tool that improves non-operative management success rates and bridges the non-operative and operative intervention procedures. Therefore, we should aim to develop a therapeutic system that can provide the additional interventions, such as fluid resuscitation by using blood transfusion, repeated TAE, and surgical intervention, for patients of any age who are haemodynamically unstable, before or after non-operative management.

### Limitation

This study has several limitations. First, it was a retrospective analysis conducted at a single centre; thus, issues of a small sample size and selection bias could not be excluded from this study. In addition, we did not include paediatric patients younger than 4 years in this study. Second, we cannot consider the influence of the additional treatments such as blood infusion, REBOA, and surgical intervention, which the patients underwent before or after TAE. Finally, we could not evaluate the long-term influence of TAE, including radiation-induced malignancies, which have been reported to occur at a higher incidence in paediatric patients undergoing CT scans than that in adults [23]. Therefore, in the future, we intend to conduct additional and detailed studies and multivariate analysis with a large cohort and longer follow-up duration to complement the limitations of this study.

## Conclusions

It is possible to provide an equal level of care related to TAE for paediatric blunt torso trauma with haemorrhage similar to what we do for adults in the trauma centre. Although TAE is an efficient and safe tool for paediatric blunt torso trauma, we should prepare alternative haemorrhage control procedures such as blood transfusion, repeated TAE, and operative management as soon as possible to prepare for patients in haemodynamically unstable states.

## Data Availability

The datasets supporting the conclusions of this article are available from the corresponding author on reasonable request.

## Abbreviations

TAE: Transcatheter arterial embolisation
YCMTC: Yokohama City Major Trauma Centre
IVR: Interventional radiology
RBC: Red cell concentrate
FFP: Fresh frozen plasma
CT: Computed Tomography
ISS: Injury severity score
RTS: Revised trauma score
TRISS: Trauma and injury severity score
ICU: Intensive care unit
SMR: Standardised mortality ratio
IQR: interquartile range
REBOA: Resuscitative endovascular balloon occlusion of the aorta
